# Pathomechanisms and Treatment Implications for Stroke in COVID-19: A Review of the Literature

**DOI:** 10.3390/life12020207

**Published:** 2022-01-29

**Authors:** Brian Stamm, Deborah Huang, Regina Royan, Jessica Lee, Joshua Marquez, Masoom Desai

**Affiliations:** 1 Department of Neurology, School of Medicine, Northwestern University Feinberg, Chicago, IL 60611, USA; deborah.huang@northwestern.edu (D.H.); jessica.lee1@northwestern.edu (J.L.); 2 Department of Emergency Medicine, School of Medicine, Northwestern University Feinberg, Chicago, IL 60611, USA; mary.royan@nm.org; 3 Department of Neurology, School of Medicine, University of New Mexico, Albuquerque, NM 87144, USA; joshmarquez@salud.unm.edu

**Keywords:** COVID-19, acute ischemic stroke, pathomechanism stroke, management of stroke, SARS-CoV-2, acute ischemic stroke, intracerebral hemorrhage, pathophysiology, pathomechanisms, treatments, cryptogenic stroke

## Abstract

Stroke in patients with COVID-19 has received increasing attention throughout the global COVID-19 pandemic, perhaps due to the substantial disability and mortality that can result when the two conditions co-occur. We reviewed the existing literature and found that the proposed pathomechanism underlying COVID-19-associated ischemic stroke is broadly divided into the following three categories: vasculitis, endothelialitis, and endothelial dysfunction; hypercoagulable state; and cardioembolism secondary to cardiac dysfunction. There has been substantial debate as to whether there is a causal link between stroke and COVID-19. However, the distinct phenotype of COVID-19-associated strokes, with multivessel territory infarcts, higher proportion of large vessel occlusions, and cryptogenic stroke mechanism, that emerged in pooled analytic comparisons with non-COVID-19 strokes is compelling. Further, in this article, we review the various treatment approaches that have emerged as they relate to the proposed pathomechanisms. Finally, we briefly cover the logistical challenges, such as delays in treatment, faced by providers and health systems; the innovative approaches utilized, including the role of tele-stroke; and the future directions in COVID-19-associated stroke research and healthcare delivery.

## 1. Introduction

The first known case of coronavirus disease 2019 (COVID-19) due to severe acute respiratory syndrome coronavirus 2 (SARS-CoV-2) was identified in Wuhan, China in December of 2019 [1]. Since that time, the medical world has rushed to identify other systemic manifestations of the virus beyond the severe acute respiratory syndrome. Neurological involvement is overall common, with approximately 40% of patients hospitalized in a major healthcare system manifesting with neurological signs or symptoms at disease onset [2]. Stroke, however, is a relatively uncommon neurological manifestation of COVID-19, occurring in 1.4% of all patients with COVID-19 in one major systematic review and meta-analysis [3]. Yet, stroke in COVID-19 has received substantial attention from both the medical and lay community, perhaps given the substantial disability that results when the two conditions co-occur and the nightmarish scenarios of young COVID-19 patients without traditional vascular risk factors presenting with large-vessel occlusions [4]. Given the relative infrequency of these cases, reports on the mechanisms of stroke in COVID-19 are largely confined to case reports and series. Several review articles have been published but given the dynamic and evolving nature of the pandemic, the topic requires frequent and comprehensive revisitation. Even though the incidence of stroke in COVID-19 patients is only 1–2%, the global scale of the pandemic ensures the relevance of the topic. In this review, we endeavor to describe state-of-the-art postulated pathomechanisms and potential therapeutic approaches to stroke in COVID-19 patients, incorporating the latest evidence and theories.

## 2. Basic Epidemiology

A recent systematic review and meta-analysis of stroke in COVID-19 patients found that 87.4% had an acute ischemic stroke (AIS) whereas 11.6% developed intracerebral hemorrhage (ICH). Patients with AIS had a median NIHSS score of 15, with large vessel occlusions (LVOs) accounting for 79.6% of cases. The relatively high proportion of LVO cases (597 out of 1189 total cases) [3] validated trends reported earlier in the pandemic [4]. Mechanistically, the majority of cases in this cohort were deemed cryptogenic (44.7%), cardioembolic (21.9%), or large vessel atherosclerotic (10.6%), whereas small vessel disease was rare (3.3%). Another theme that emerged was that COVID-19-associated strokes often had multiple arterial territories affected [3]. COVID-19 stroke patients with ICH frequently presented with lobar hemorrhage (44.1%) and herniation (18.5%).

### Comparison between COVID-19-Associated and Non-COVID-19 Strokes

The same systematic review and meta-analysis mentioned above utilized 11 studies to compare stroke patients with and without COVID-19 infection. The authors found that patients with stroke and COVID-19 were younger and more commonly male. COVID-19 stroke patients were less likely to have the most significant risk factor for stroke, hypertension, and less frequently had a prior history of stroke. However, both cohorts had similar rates of other traditional vascular risk factors, including atrial fibrillation, coronary artery disease, smoking, dyslipidemia, and diabetes mellitus. As might be expected given the epidemiology presented above, COVID-19 cases of stroke had: a higher proportion of large vessel occlusions, higher stroke severity, and more frequent cryptogenic stroke than non-COVID-19-associated strokes. Concerning outcomes, COVID-19 stroke patients had higher in-hospital mortality rates even after receiving acute stroke interventions (e.g., tPA and thrombectomy) with similar frequency to stroke patients without the infection [3]. Other large systematic reviews have been in agreement with these findings, drawing similar comparisons between the characteristics and outcomes of patients with COVID-19-associated and non-COVID-19 strokes [5]. Table 1 lists some representative data from various studies comparing COVID-19 and non-COVID-19-associated strokes [6,7,8].

## 3. Biological Mechanisms

At the outset, it is important to consider that COVID-19 infection and stroke may have an incidental rather than causative association given the overall frequency of the virus in the general population. For example, some patients with COVID-19 may develop strokes due to their a priori, longstanding vascular risk factors, such as hypertension or atherosclerosis, which may have little or no direct relationship to their SARS-CoV-2 infection. Indeed, a large observational study of 844 hospitalized COVID-19 patients demonstrated that the majority of patients who developed strokes in their cohort had traditional vascular risk factors, and conventional stroke mechanisms were common [9]. However, given that certain unique characteristics of COVID-19-associated strokes emerged in pooled analyses (such as multivessel territory infarcts, overrepresentation of large vessel occlusions, and cryptogenic stroke mechanism) [3], it can be postulated that certain biological mechanisms are at play in COVID-19 infection that drive these particular stroke phenotypes. Additionally, controlling against other viruses, such as influenza, SARS-CoV-2 infection has a 7.6-fold higher odds of developing stroke [10]. Additionally, there have been cases reported of AIS as the primary presenting symptom of SARS-CoV-2 infection [11] or early in the disease course without prominent respiratory symptoms [12], again suggesting that there may be a biological basis driving these findings.

Viral infection has been shown in both clinical and preclinical studies to be an independent stroke risk factor, most notably with influenza and herpesviruses [13]. Based on the high number of case reports and myriad of neurologic symptoms associated with COVID-19, one would expect this to also be the case with SARS-CoV-2. However, current preclinical models do not demonstrate a clear association between COVID-19 disease and stroke risk. Early mouse models recapitulated only mild patterns of COVID-19 disease [14,15], but the development of humanized angiotensin-converting enzyme 2 (ACE2) transgenic mice allowed the study of more severe SARS-CoV-2 pulmonary infections [15]. Studies of pathogenicity and pulmonary infection have now been established in non-human primates, ferrets, cats, and dogs [14,16]; yet, there have been few reports of cerebrovascular disease-specific findings.

Studies in ACE2 transgenic mice support a neurotropism of the SARS-CoV-2 virus, including invasion of the olfactory bulb and spread to multiple areas in the brain [17]. Neurotropism has also been demonstrated with other coronaviruses [17,18]. In an analysis of astrocytes from SARS-CoV-2 infected mice, Netland et al. discovered significant upregulation of IL-6, a cytokine specifically implicated in multiple pathomechanisms of COVID-19-associated stroke [19]. Recent mouse studies of large vessel stroke, via middle cerebral artery occlusion, demonstrate increased ACE2 expression in ischemic brain tissue, as well as in endothelial cells of diabetic and cigarette smoke-exposed mice [20]. The study’s authors postulate that increased ACE2 expression may partially explain the higher infection risk and more severe presentation of COVID-19 disease observed amongst individuals with diabetes and/or smoking history. However, these findings do not add to our understanding of the relationship between ACE2, COVID-19, and stroke risk. The pathomechanisms invoked in COVID-19-associated stroke remain to be proven in preclinical studies.

### 3.1. Mechanisms of Acute Ischemic Stroke in COVID-19 Patients

**Figure 1:**
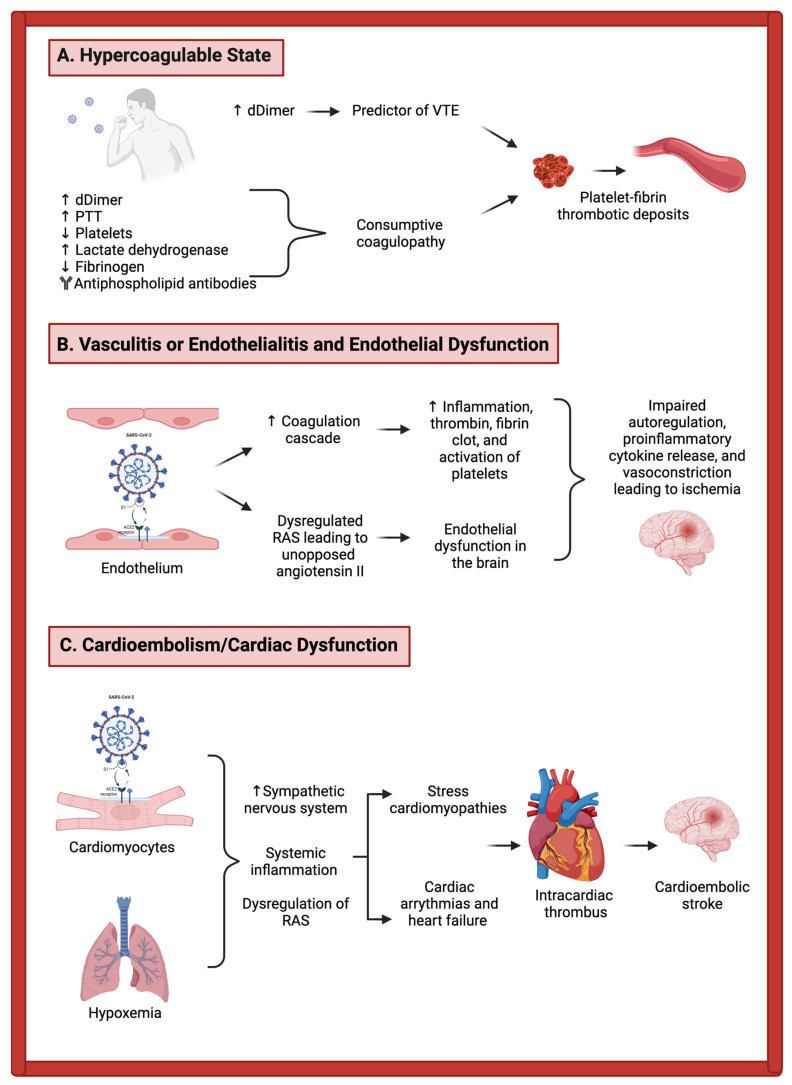
Proposed pathomechanisms of acute ischemic stroke in COVID-19 infection. (**A**) Hypercoagulability in COVID-19 is presumed to be caused by an increase in inflammatory and procoagulant markers that lead to VTE and/or platelet-fibrin thrombotic deposits. (**B**) Endothelial dysfunction/endothelialitis involves SARS-CoV-2 interaction with the ACE2 receptor on the endothelial cells. This interaction leads to an inflammatory response, upregulation of the coagulation cascade, and dysregulation of the renin–angiotensin system (RAS), which may cause thrombosis, fibrin clot formation, and vasoconstriction. (**C**) Similarly, SARS-CoV-2 infection via ACE2 receptor on the cardiomyocytes leads to dysregulation of the RAS and inflammation. This mechanism in conjunction with hypoxemia may lead to stress cardiomyopathies, cardiac arrhythmias, and heart failure, increasing the possibility of an intracardiac thrombus. All three of these proposed mechanisms increase the possibility of vessel obstruction that may lead to brain ischemia. Created with BioRender.com (accessed on 20 January 2022).

The described potential pathological mechanisms in COVID-19-associated ischemic stroke fall largely within the following three overall categories: vasculitis, endothelialitis, and endothelial dysfunction; hypercoagulable state; and cardioembolism secondary to cardiac dysfunction [21,22,23]. These mechanisms are depicted in Figure 1.

#### 3.1.1. Vasculitis, Endothelialitis, and Endothelial Dysfunction

The mechanism by which SARS-CoV-2 enters the alveolar epithelium is by interaction with the ACE2 receptor which, along with local inflammatory effects, leads to a massive downstream inflammatory response with the release of cytokines and chemokines, ultimately activating epithelial cells, monocytes, and neutrophils. Importantly, vascular endothelial cells also contain ACE2 receptors and may also be directly infected, which can lead to impaired function in the endothelium and upregulate the coagulation cascade. This direct invasion by the virus of the endothelial cells can result in endothelial inflammation, referred to as “endothelialitis”, one of the proposed major mechanisms of thrombosis during COVID-19 infection [24].

In addition to the above mechanisms, when SARS-CoV-2 binds to the ACE2 receptor, this can lead to the receptor itself becoming endocytosed, which has downstream effects on the renin–angiotensin system (RAS) [15]. The usual function of ACE2—the conversion of angiotensin II to angiotensin-(1,7)—thus becomes compromised, leading to an upregulation of angiotensin II [15,25]. This unopposed angiotensin II can lead to worsened endothelial dysfunction in the multiple organ systems in which these receptors are expressed, including the brain. This endothelial dysfunction can ultimately lead to the formation of thrombin and fibrin clots at the surface of the endothelium, which can predispose to thromboembolism [25]. Platelets become activated, as well as the protease-activated receptor signaling pathway, leading to further inflammation. The co-occurrence of thrombosis and inflammation shifts the balance towards a highly inflammatory cycle [26]. The downstream effects include impaired autoregulation, proinflammatory cytokine release [27], and vasoconstriction leading to ischemia [28].

The above mechanisms involving direct endothelial damage by the virus and more systemic downstream inflammatory responses have been previously demonstrated in cases that underwent pathology review. For instance, in a pathological study of patients (two autopsies plus one surgical biopsy) with COVID-19 infection, the investigators found viral inclusions within the endothelial cells of various organs, including heart, lungs, small bowel, and kidneys, with resultant apoptosis. The authors postulated that multiorgan failure in severe cases of SARS-CoV-2 infection could potentially be explained by this widespread endothelialitis [24]. Even more recently, a group published a series of six cases, which were all positive for SARS-CoV-2 infection and had cerebral petechial hemorrhages and microthrombi at the time of autopsy. Two of the patients were found to have cerebrovascular intramural inflammatory infiltrates, with associated increased levels of SARS-CoV-2 receptor ACE2 within the cerebral blood vessels. These findings were deemed representative of neuro-pathologically confirmed SARS-CoV-2-associated endothelialitis [29].

#### 3.1.2. Hypercoagulability

Coagulopathy is a common occurrence in SARS-CoV-2 infection, with prior reports suggesting that up to 20–55% of hospitalized COVID-19 patients will have laboratory values consistent with coagulation pathway activation, including elevated D-dimer, prolongation of prothrombin time, and thrombocytopenia. These inflammatory markers can correlate with the risk of critical illness, with a more than linear relationship between D-dimer levels and the likelihood of critical illness [30], and clinicians throughout the pandemic have used these levels to risk-stratify their patients. Additionally, elevated D-dimer results can predict venous thromboembolism (VTE) events during COVID-19 infection, with one study of critically ill patients with COVID-19 showing that D-dimer levels greater than 1500 ng/mL had a sensitivity of 85.0%, specificity of 88.5%, and negative predictive value of 94.7% for VTE [31]. This coagulopathy in the setting of critical illness is not specific to COVID-19 infection and has been seen in other infectious-related pro-inflammatory states and has been called “sepsis-induced coagulopathy”. Elevated D-dimer levels have additionally been seen in patients with COVID-19 and stroke, and this so-called sepsis-induced hypercoagulable state has been postulated to contribute mechanistically to the development of stroke in COVID-19 patients [15,32] and patients with other infectious and inflammatory states [33].

Though similar to mechanisms previously described in sepsis, COVID-19-associated coagulopathy (CAC) is distinct given its prominence of inflammatory cytokines and complements [34,35]. In addition to these inflammatory markers, COVID-19 patients with multi-hemispheric infarcts have been found to have antiphospholipid antibodies, including anticardiolipin and anti-β-glycoprotein I antibodies [36], again distinguishing CAC from the coagulopathy seen in sepsis. In patients with severe SARS-CoV-2 infection, the overall inflammatory profile can resemble that seen in disseminated intravascular coagulation (DIC), a so-called consumptive coagulopathy, with thrombocytopenia, prolonged prothrombin time, elevated D-dimer and lactate dehydrogenase, and decreased fibrinogen. At autopsy, these patients have multiorgan microvascular platelet-fibrin-rich thrombotic deposits [24,37]. The hypercoagulable state can lead to strokes by several different mechanisms, including paradoxical embolism from lower extremity VTE to the brain through a patent foramen ovale, marantic endocarditis, cerebral venous sinus thrombosis, among others [22,38].

#### 3.1.3. Cardiac Dysfunction

There is a multitude of ways that COVID-19 infection can lead to cardiac dysfunction, subsequently leading to elevated risk for cardioembolic stroke [39,40]. Myocardial injury is relatively common in the infection, occurring in >7% of cases (and >20% of critically ill cases) [41]. Similar to the direct invasion of SARS-CoV-2 within endothelial cells via the ACE2 receptor interaction, cardiomyocytes contain ACE2 receptors and are thus at risk of direct invasion by the virus. Again, similar to the pathology of other systemic organ systems, coronavirus-associated myocarditis is thought to be secondary to the effects of direct invasion plus the subsequent systemic inflammatory cascades [42]. Other potential effects of SARS-CoV-2 on the cardiovascular system include stress cardiomyopathies secondary to hypoxemia and upregulation of the sympathetic nervous system, due to the dysregulation of the RAS, as described previously. Cardiac arrhythmias and heart failure may develop for similar reasons [43]. The development of any of these cardiac complications from SARS-CoV-2 infection can ultimately lead to intracardiac thrombus, thus elevating the patient’s risk for cardioembolic stroke mechanism [21].

#### 3.1.4. Overlap between Mechanistic Categories

Finally, there is some overlap between the mechanistic categories delineated above. For example, the pro-inflammatory cytokine storm that results from the upstream interaction between SARS-CoV-2 and the ACE2 receptor at the level of the endothelium ultimately leads to a hypercoagulable state. This is because there is an interaction between cytokines and procoagulant factors that play a role in the coagulation pathway. For example, IL-1, IL-6, and TNF-α help promote tissue factor secretion, which then helps stimulate the extrinsic coagulation system. These cytokines also help express PAI-1, which ultimately inhibits the fibrinolysis pathway [44,45]. Thus, these mechanistic categories should not be thought of in isolation, but rather as interactive systems with combined relevance for the final common pathway of AIS in COVID-19 infection.

Additionally, no one mechanistic category alone clearly explains the COVID-19-associated stroke phenotype in a unified fashion. While the microthrombi formation in the setting of a consumptive coagulopathy described above could explain the multivessel territory infarcts seen in COVID-19-associated stroke, it does not clearly explain the seemingly higher proportion of LVOs. Given that a higher-than-expected proportion of patients who developed LVOs with concomitant COVID-19 infection were relatively young, without traditional vascular risk factors, in situ thrombosis secondary to plaque rupture seems relatively unlikely to be the mechanism of LVO in this population [21]. Rather, it seems more plausible that the hypercoagulable state leads to venous thromboembolism with paradoxical embolism or cardioembolism, akin to the final pathway stroke mechanism seen in hypercoagulability of malignancy [46]. The various mechanisms of resultant cardiac dysfunction described in COVID-19 infection could also partially explain the seemingly overrepresented cases of LVO.

### 3.2. Mechanisms of Intracerebral Hemorrhage in COVID-19 Patients

Overall, the incidence of intracerebral hemorrhage (ICH) in COVID-19 infection is much lower than acute ischemic stroke, and ICH is generally thought to be a rare complication of COVID-19. In a review of ICH and COVID-19 that included 36 original studies, only 188 cases of ICH were found [47]. Another review found 148 cases of intracranial hemorrhage across 23 studies, with a pooled incidence of 0.7%, with half of the patients on anticoagulation [48]. While some of the following COVID-19-related pathomechanisms may contribute to the development of ICH, the causative link is even less clear than in ischemic stroke, given that many cases of ICH in COVID-19 patients also have concomitant risk factors for ICH related to their critical illness, such as treatment with anticoagulation and admission to intensive care unit (ICU) settings, where various treatment modalities, such as extracorporeal membrane oxygenation (ECMO), can elevate the risk of ICH [49]. Even given its relative rarity, ICH in COVID-19 infection is a highly important entity for clinicians to recognize early and prevent, given that multiple prior studies have reported its mortality rate at approximately 50% [47,48].

Similar to the mechanisms proposed for ischemic stroke in COVID-19 patients, the potential pathomechanisms in ICH take the form of direct and indirect injuries to the endothelium [47,50]. The direct invasion of the endothelial cells via ACE2 receptors, as described above, can cause cell death and can lead to vessel compromise and ultimately rupture [50]. Indirectly, this initial interaction of the virus with ACE2 receptors leads to a massive inflammatory cascade of cytokine and chemokine release, which may potentially lead to a breakdown in the blood–brain barrier with disruption of tight junctions [51,52], thus elevating the risk of ICH. Additionally, the consumptive coagulopathy pathophysiology discussed above elevates risk not only for ischemic but also for hemorrhagic events [53].

Finally, direct invasion of the ACE2 receptor ultimately leads to the downregulation of ACE2 receptors, which can influence the development of ICH in multiple ways [51]. Importantly, such downregulation can increase local levels of angiotensin II, which can lead to resultant hypertension [54]. This mechanism may particularly exacerbate the risk of ICH in COVID-19 patients with preexisting comorbidities [51].

**Figure 2:**
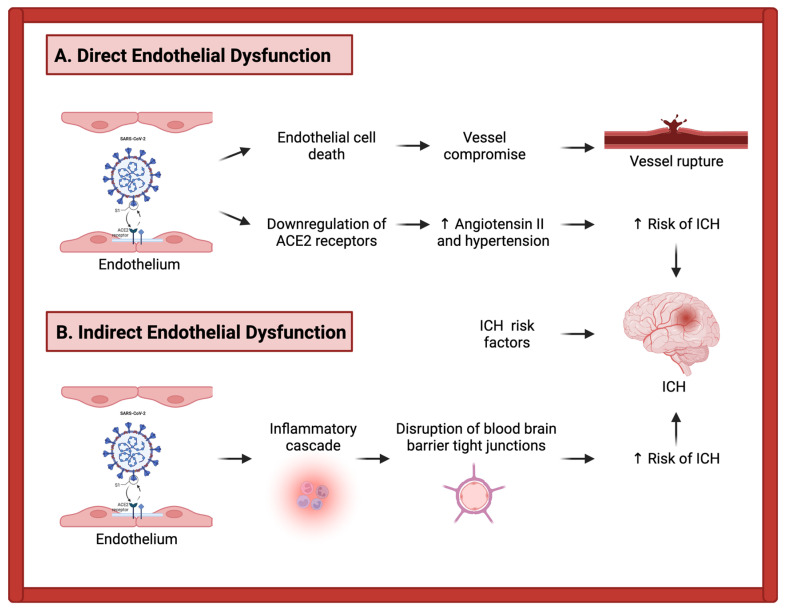
Proposed pathomechanisms of intracerebral hemorrhage (ICH) in COVID-19 infection: direct and indirect pathways involving endothelial dysfunction. (**A**) Direct pathway: SARS-CoV-2 invasion of endothelial cells through ACE2 receptors may lead to endothelial cell death, vessel dysfunction, and rupture. Downregulation of ACE2 receptors may increase angiotensin II levels, resulting in hypertension and increased ICH risk. (**B**) Indirect pathway: SARS-CoV-2 interaction with endothelial ACE2 receptors leads to cytokine and chemokine release. This inflammatory response can interrupt tight junctions within the blood–brain barrier, increasing ICH risk. In addition to the direct and indirect pathways, ICH risk is increased by other risk factors such as anticoagulation, ICU admission, consumptive coagulopathy, and preexisting comorbidities. Created with BioRender.com (accessed on 20 January 2022).

Though the mechanisms just described may inherently contribute to the development of ICH in COVID-19 patients, there are multiple external factors that many of these patients may also have concerning their critical illness and its treatment, which may concurrently elevate the risk of hemorrhage. For example, both therapeutic anticoagulation and mechanical intubation are risk factors for the development of ICH in COVID-19 infection [49,55,56]. Such treatment modalities are common in COVID-19 infection, particularly when the severity of illness warrants ICU admission, which poses a challenge to treating providers of balancing the risks and benefits of their approaches. These discussed pathomechanisms of ICH in COVID-19 infection are depicted in Figure 2.

## 4. Case Illustration

**Figure 3:**
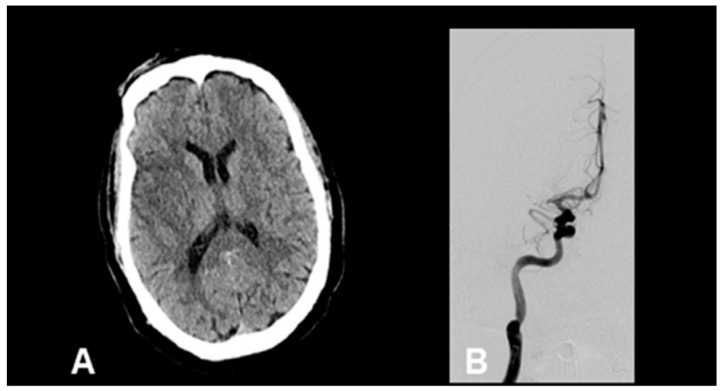
Representative neuroimaging from a patient with recent SARS-CoV-2 infection, new-onset atrial fibrillation, and proximal right MCA occlusion. (**A**) Non-contrast head CT reveals areas of hypoattenuation within the right MCA vascular territory. (**B**) Conventional angiography revealing persistent proximal M1 occlusion. Adapted with permission from Batra, A.; Clark, J.R.; LaHaye, K.; Shlobin, N.A.; Hoffman, S.C.; Orban, Z.S.; et al. (2021) [57].

A 66-year-old male with a history of hypertension and asthma initially presented with fevers, chills, nausea, vomiting, and diarrhea. He tested positive for SARS-CoV-2 at that time and was discharged home. Six weeks later, he presented to the hospital with similar symptoms and was hospitalized. On hospital day three, he was noted to have new-onset atrial fibrillation. He was subsequently noted to have acute left hemiparesis with an NIH stroke scale of 15. CT brain showed substantial hypodensity in the right middle cerebral artery (MCA) territory and CT angiography demonstrated a proximal right M1 MCA occlusion (Figure 3A). Additional findings on angiography showed non-calcified atherosclerosis with <10% stenosis of carotid bulbs and a normal aortic arch. The patient was not a candidate for pharmacologic thrombolysis given that recognition of his symptoms was outside the window for treatment, however, CT perfusion suggested a mismatch profile favorable for mechanical thrombectomy. Multiple attempts to recanalize the MCA occlusion were unsuccessful even after the use of eptifibatide infusion and multiple passes with an aspiration catheter (Figure 3B). Transthoracic echocardiography (TTE) was also obtained on day three of admission which showed Left Ventricular Ejection Fraction (LVEF) of 28% (unknown baseline), LV dilation, no shunt, and no thrombus. TTE was repeated on day five of admission which showed LVEF of 19%, severe Right Ventricular (RV) dysfunction, no shunt, and no thrombus. Case reproduced with permission from Batra, A.; Clark, J.R.; LaHaye, K.; Shlobin, N.A.; Hoffman, S.C.; Orban, Z.S.; et al. (2021) [57].

This case demonstrates several of the proposed mechanisms for acute stroke in those recovering from SARS-CoV-2 infection, namely new-onset cardiac dysfunction with the development of atrial fibrillation and depressed EF in a patient with few pre-existing comorbidities. The development of such cardioembolic stroke mechanisms may partly explain the oversampled number of large vessel occlusions seen in SARS-CoV-2 infection. Additionally, the case illustrates the challenges encountered in the treatment of COVID-19-associated strokes.

## 5. Delays in Care

In the early months of the pandemic, many emergency departments saw a net decrease in the volume of patients, including presentations of many acute conditions [58,59,60]. Multiple studies have shown delays in the presentation of acute stroke during the pandemic including periods where localities had stay-at-home orders in place [61,62,63]. Altschul and colleagues found that in the first two months of the pandemic, patients who presented with LVO were younger (66 vs. 72 years, *p* < 0.061), had lower ASPECTS (7 vs. 9, *p* < 0.001), and took longer to arrive at the hospital (361 vs. 152 min, *p* < 0.004) with no other major differences compared to patients presenting with LVO preceding the pandemic [61]. Challenges in the timely presentation and diagnosis of a stroke may necessitate the increased use of advanced imaging such as CT perfusion (CTP) or Magnetic Resonance Imaging (MRI) to determine a patient’s candidacy for therapeutic intervention [22]. While pre-hospital delays are likely the largest contributor to the decrease in strokes amenable for intervention [64], delays in care once the patient has arrived at the hospital due to resource limitations and infection control likely also play a factor [65]. Some of the unique challenges faced in diagnosing, treating, and managing COVID-19-associated stroke are listed in Table 2.

## 6. Potential Treatment Options

We will focus mostly on the treatment of acute ischemic stroke (AIS) in the following discussion, though we acknowledge that hemorrhagic stroke represents an important portion of cerebrovascular events seen with COVID-19. However, management of intracerebral hemorrhage often requires escalation to the ICU, a topic outside our scope of coverage. Even though AIS associated with COVID-19 may result from multiple new pathomechanisms described above, the treatment of AIS has remained largely the same [22].

### 6.1. IV Thrombolysis

Patients with AIS presenting within the IV thrombolysis time window should receive alteplase or tenecteplase, whichever is the approved standard of care at the treating facility. Some institutions made the switch from alteplase to tenecteplase before the COVID-19 pandemic, in the treatment of AIS. Warach et al. propose that the benefits of tenecteplase over alteplase are particularly salient in the COVID-19 era due to faster infusion time of tenecteplase, which frees the patient’s intravenous (IV) access (tenecteplase requires only a five-second IV infusion, compared to 60+ minutes for alteplase), and decreases exposure of healthcare providers to SARS-CoV-2 infected individuals [66]. Furthermore, there have been worldwide shortages of alteplase since the suggestion that alteplase administration may be beneficial in acute respiratory distress syndrome (ARDS) treatment [66]. After IV thrombolytic administration, providers should continue to monitor hemodynamics (goal blood pressure < 185/<110 mmHg), trend neurologic examinations, and stay vigilant for adverse effects of thrombolytic therapy—these clinical practices remain the same regardless of SARS-CoV-2 infection status. Providers must be aware that SARS-CoV-2 infected patients are at high risk of continued thrombotic events due to the pathomechanisms mentioned earlier [22].

### 6.2. Mechanical Thrombectomy

Large vessel occlusion (LVO) represents a higher proportion of acute ischemic strokes in SARS-CoV-2 infected versus non-infected patients [3,4], prompting discussions about the logistical challenges of mechanical thrombectomy (MT) in COVID-19 patients. At the beginning of the pandemic, arising from significant concerns about the high transmissibility of SARS-CoV-2 and the need for aerosol-generating procedures in the stabilization of infected stroke patients, Khosravani et al. proposed the creation of a “Protected Code Stroke” pathway to protect providers yet maintain a streamlined process for acute stroke evaluation in patients with unknown or positive infection status [65]. Now, over two years from the start of the COVID-19 pandemic, institutional processes for the safe and timely evaluation of patients continue to evolve and improve.

Neurointerventionalists face unique challenges in the MT of COVID-19 LVO strokes, likely resulting from the pathomechanisms described earlier [22]. In a French case series of 10 SARS-CoV-2 infected patients who underwent MT for acute large vessel occlusion stroke, half of whom received IV recombinant tissue-plasminogen activator (rt-PA) pre-thrombectomy, successful reperfusion was achieved in nine. None of the recanalizations, however, were achieved on the first-pass [67]. Additionally, 4 of the 10 patients developed arterial re-occlusion less than 24 h post-procedure. Multi-vessel territory, re-occlusion, clot fragmentation, and high clot burden in COVID-19 LVO patients, reduce the efficacy of MT [3,68]. Papanagiotou et al. note that MT treatment of COVID-19 strokes may require the employment of techniques utilized in LVOs resulting from cancer-associated coagulopathy, sepsis, and disseminated intravascular coagulation [69]. This includes strategies such as administering intraluminal fibrinolytic or antiplatelet agents, multiple passes for clot retrieval, and stent deployment [68].

Additionally, there was a lower number of cases receiving MT during the pandemic [70]. There could be several factors responsible for this including delays in the transfer of patients to tertiary care centers, testing of COVID-19 required by certain institutions before receiving MT, apprehension of patients which prevented them from seeking care and overwhelmed healthcare systems, to name a few.

Some of us have cared for COVID-19 positive patients who developed recurrent strokes, either during the same hospital admission or returning after discharge with new stroke symptoms. However, the current literature is mixed on the true rate of recurrent stroke in patients with COVID-19. In one retrospective study in Ghana, the recurrent stroke rate increased almost two-fold to 19.0% in 2020, from 10.9% in 2019 (adjusted odds ratio 1.22), coinciding with the rise in COVID-19 hospitalizations [71]. Other centers have described a rate of arterial re-occlusion and recurrent stroke between 10 and 40% [68,72]. In contrast, a multicenter case–control study in the United Kingdom that included 86 strokes from 13 different institutions revealed no statistically significant increase in stroke recurrence rate in COVID-19 positive vs. negative patients (2.3% vs. 1.0%, respectively) [73].

### 6.3. Therapeutic Anticoagulation

The following pathomechanisms highlight the reasoning for therapeutic anticoagulation in some COVID-19 patients: hypercoagulability from a sepsis-induced and/or consumptive coagulopathy, microthrombi formation, endothelial dysfunction, and cardiac dysfunction predisposing to cardioembolic events. However, what patients truly benefit from therapeutic anticoagulation? Should anticoagulation be initiated on primary prevention (ex: upon diagnosis of COVID-19 or by proxy of inflammatory markers) or secondary prevention (i.e., post-thromboembolic event) basis?

Outside of the stroke population, there remains ongoing debate about the use of therapeutic anticoagulation in COVID-19 patients. Earlier this year, a French multi-center study of 25 hospitals and 2878 consecutive patients found that pre-hospitalization oral anticoagulation use was a protective factor in COVID-19 patients, with an adjusted hazard ratio of 0.43 (95% CI, 0.29–0.63; *p* ≤ 0.001) for ICU admission and 0.76 (95% CI, 0.61–0.98; *p* = 0.04) for ICU admission or in-hospital mortality [74]. Other studies have also demonstrated decreased mortality in severe COVID-19 patients who were initiated on anticoagulation [75]. However, findings are mixed in multiple, large, randomized control trials (RAPID, INSPIRATION, as well as a multiplatform adaptive-design trial incorporating the REMAP-CAP, ATTACC, and ACTIV-4A networks). The multiplatform trial demonstrated a reduction in venous thromboembolic events (VTE) and better odds of being alive and organ-support free in the therapeutically anticoagulated group at 21 days in non-ICU hospitalized COVID-19 patients, but the study was stopped early for futility in patients requiring ICU-level care, who were randomized to therapeutic versus prophylactic anticoagulation, with no reduction seen in the anticoagulated group in terms of organ-support needs or death at 21 days [76]. The American Society of Hematology currently recommends that critically-ill COVID-19 patients not be routinely started on therapeutic anticoagulation without clear evidence of VTE; and for moderately ill patients not requiring ICU-level care, that providers consider therapeutic anticoagulation based on the individual’s perceived clinical benefit in the absence of VTE [76]. COVID-19 patients with VTE should receive low molecular weight heparin (LMWH) or unfractionated heparin (UFH) treatment appropriate for their VTE. The role of oral anticoagulants in the treatment of COVID-19 thromboembolic complications has not been well-established [22]. The National Institutes of Health COVID-19 treatment guidelines also recommend against the routine use of therapeutic anticoagulation in patients who lack a clear indication [77]. All patients with low bleeding risk should still receive thromboprophylaxis with LMWH or UFH.

In the AIS population, the decision to initiate anticoagulation deserves even more caution due to the risk of hemorrhagic transformation. Stroke care guidelines have not modified recommendations on anticoagulation use in COVID-19 stroke patients [78]. The same approach to post-stroke care is to be applied regardless of SARS-CoV-2 infection status. With regards to therapeutic anticoagulation, patients should not be empirically initiated on therapeutic anticoagulation unless the bleeding risk is low and they have clear evidence of VTE or risk factors such as atrial fibrillation [78]. Even then, the timing of anticoagulation initiation should be carefully chosen and postponed for when the patient’s bleed risk is acceptably low. Thromboprophylaxis at the time of admission in patients with low bleed risk, or the 24-h mark after IV thrombolysis and no evidence of bleeding, remains the standard of care [79].

Thorough investigations for clear evidence of VTEs, other causes of hypercoagulability, and cardiac dysfunction are particularly important to pursue in COVID-19 stroke patients, for appropriate treatment guidance. Data remains limited regarding the anticipated duration of anticoagulation in COVID-19 stroke patients [22]. Antiplatelet and statin therapy, as well as modification of vascular risk factors, should be pursued according to the current standards of care. Interestingly, stroke occurrence in COVID-19 positive patients who were already on therapeutic anticoagulation, either for prior stroke or other thromboembolic events, has also been described [80]. As we continue to learn more about the pathomechanisms underlying COVID-19-associated coagulopathy and the heterogeneity of its presentations, we fully anticipate the best practice guidelines regarding anticoagulation in COVID-19 stroke care to continue evolving as well.

### 6.4. ACE-Inhibitors and Angiotensin Receptor Blockers

After the SARS-CoV-2 interaction with ACE2 receptors was discovered, there was initial controversy about whether ACE-inhibitor (ACE-I) and Angiotensin receptor blocker (ARB) usage upregulate the number of ACE2 receptors in a way that increases susceptibility to SARS-CoV-2 infection [81]. This is now widely accepted to not be the case, after the publication of multiple observational studies showing no detriment with ACE-I/ARB therapy [82]. A randomized control trial (BRACE CORONA) was published in early 2021 that showed no difference in survival amongst patients already on ACE-Is/ARBs who were randomized upon hospitalization with COVID-19 to continue or stop the antihypertensive therapy [83,84]. New studies describe lower SARS-CoV-2 infection rates with ACE-I therapy, though this benefit has not held for other clinical outcomes such as ICU admission, mortality, and ventilator-free days [82,85]. Our takeaway is that, in stroke patients who are on ACE-I or ARB for hypertension, COVID-19 positivity is not a reason to withhold therapy.

### 6.5. Experimental Therapies in Ongoing Trials

With the scale of this pandemic and our understanding of the SARS-CoV-2 pathomechanisms so far, it is perhaps not surprising that clinical studies have exploded in number over the past year. These studies span the diagnostic, therapeutic, and rehabilitative spectrums. Within therapeutics, experimental therapies under study include inhaled APN01, a recombinant ACE2 enzyme to block SARS-CoV-2 binding of ACE2 receptors [15,86], antivirals previously used in the treatment of HCV and influenza [87,88], convalescent plasma, intravenous immunoglobulins, and even estrogen therapy. It will be interesting to learn from the successes and failures of these and other experimental therapies, including their impact on stroke risk and outcomes.

## 7. Ancillary Treatment Approaches and Other Considerations

### 7.1. Tele-Stroke

The management of stroke patients throughout all stages of their care has been significantly impacted by disruptions in healthcare delivery during the COVID-19 pandemic. Tele-medicine was widely adopted during the early months of the pandemic to mitigate these disruptions while minimizing contagion risk between patients and healthcare providers [89]. At the federal and state levels, various policy changes have been made to help build or expand existing infrastructure for tele-medicine systems. Pre-dating the pandemic, tele-stroke networks in the United States have been well-established and demonstrated to improve access to critical interventions such as IV TPA and endovascular therapy. Further study is ongoing to identify the extent to which virtual care can be provided to stroke patients outside of initial tele-stroke evaluation; for example, within the hospitalized inpatient setting [90].

Interestingly, various studies have identified changes in tele-stroke utilization during the early pandemic. The most common finding has been a reduction in total tele-stroke activations, which can be at least partly attributed to reduced patient volumes. While the cause of this is likely multifactorial, it reflects a continued need to raise public health awareness of prehospital identification of stroke-like symptoms, as well as an understanding that these symptoms may constitute a medical emergency that warrants hospital presentation even during a pandemic [89,91]. Delay to treatment remains a leading cause of post-stroke morbidity and mortality and targeting both public awareness to reduce time to hospital presentation and expanding tele-stroke networks to improve time to treatment remains a priority for stroke care moving forward.

### 7.2. Rehabilitation and Prevention of Secondary Stroke

Post-stroke rehabilitation plays a major role in improving functional outcomes after acute ischemic stroke. Unfortunately, physical and occupational therapy services have been made less accessible to stroke patients throughout the COVID-19 pandemic—particularly as many patients and families opt for discharge home over acute or subacute rehabilitation facilities. Options for at-home or virtual neurorehabilitation therapies include home-based exercise regimens, which are non-inferior to outpatient therapy in select patient populations [92]. Mirror therapy can also be adapted for home use, improving motor function with minimal need for in-person therapist supervision. Finally, equipment such as TENS devices can be used at home to maintain muscle strength and reduce post-stroke spasticity [93]. Further study of functional outcomes concerning post-stroke rehabilitation settings and services may help clarify the efficacy of virtual therapy, and potentially develop more standardized treatment regimens for these patients.

## 8. Future Directions

The COVID-19 pandemic has been humbling for all of us. At the same time, the scientific community has uncovered disease pathomechanisms and tested multiple treatments at a remarkable speed. From the discovery of SARS-CoV-2 interaction with ACE2 receptors to the finding of endothelial dysfunction upregulating the coagulation cascade, as well as the cardiac dysfunction resulting from direct viral invasion and RAS dysregulation, we have come a long way from the early unknowns of the pandemic. However, our work is not yet done. Stroke in COVID-19 leads to significant morbidity and mortality [94], as illustrated in our case example. The truly wide spectrum of disease severity presents a major challenge to providers and investigators alike. How do we predict which patients might develop severe neurologic complications such as LVO stroke? Perhaps in the future, we will be able to better predict who is at risk for COVID-19-associated stroke, triage them to closer monitoring in the outpatient and inpatient settings, and prevent altogether the occurrence of stroke—or at the very least, minimize pre-hospital delays in seeking treatment. We remain hopeful that treatment delays will continue to decrease as a result of heightened awareness about the challenges in COVID-19 stroke evaluation and treatment, streamlined protocols that are the result of cumulative experience from first-line providers and interventionalists, and continual technological advancements. Lastly, and perhaps most saliently, more patients are surviving from COVID-19 disease and saddled with the sequelae of prolonged hospitalizations and complications such as stroke. This will lead to increasing demand for post-acute care and rehabilitation. The rise of tele-medicine and at-home virtual rehabilitation is starting to address this growing need, but the care of patients after the COVID-19 stroke may prove to be just as heterogeneous as the disease itself. More attention is needed on the important issue of post-stroke care in the era of COVID-19. We hope that this review provides a useful summary of our current knowledge of pathomechanisms leading to COVID-19 stroke, an important comorbidity of SARS-CoV-2 infection that has gained significant public attention.

## Figures and Tables

**Table 1:** Comparison of COVID-19 and non-COVID-19 associated strokes.

	COVID-19 Associated Stroke	Non-COVID-19 Associated Stroke
Age at stroke onset mean ± SD	65.9 ± 14.3 for all strokes 59 ± 13 for LVOs	66.7 ± 15.5 for all strokes 73 ± 18 for LVOs
Pathomechanisms	Predominant ischemic stroke mechanisms include: hypercoagluable state, endothelial dysfunction/endothelialitis, and cardiac dysfunction	Prior classication systems for ischemic stroke mechanisms have been published, e.g. TOAST, which includes: large artery atherosclerosis, cardioembolism, small vessel occlusion, stroke of other determined etiology or undetermined etiology
Acute treatments		
IV thrombolysis	Alteplase or tenecteplase, logistical benefits of tenecteplase due to faster infusion times & decreased exposures	Either alteplase or tenecteplase, whichever is the approved standard of care at the treating facility
Endovascular thrombectomy	High proportion of LVOs, and EVT is standard of care, but particular challenges arise in COVID-19 patients, including: multi-vessel territory infarcts, re-occlusions, clot fragmentation, and high clot burden	Standard of care for LVOs presenting within the treatment window
Outcomes		
LOS, mean ± SD	17.4 ± 14.8 days	8.0 ± 6.4 days
Requiring ICU care	58.7%	44.7%
In-hospital death	33%	12.9%

Abbreviations: SD: standard deviation; LVO: large vessel occlusion; IV: intravenous; EVT: endovascular thrombectomy; LOS: length of stay; ICU: intensive care unit.

**Table 2:** Challenges in diagnosing, treating, and managing COVID-19-associated stroke.

	Potential Treatment/Care	Challenges
Prehospitalization/diagnosis	Timely presentation and diagnosis with proper stroke workup, available resources, and staff trained to efficiently handle infection control	Stay at home orders during pandemic Pre-hospital visit delays (likely largest contributor) Delays in care due to resource limitations and infection control Limited use of imaging in infected patients
Hospitalization	IV Thrombolysis	Availability and infusion time of alteplase versus tenecteplase Monitoring hemodynamics Trending neurologic examinations Staying vigilant for adverse effects of thrombolytic therapy Patients at high risk of continued thrombotic events
	Mechanical Thrombectomy	General versus monitored anesthesia Recanalizations not always achieved on the first-pass Arterial re-occlusion–reducing the efficacy of MT Recurrent stroke
	Therapeutic Anticoagulation	No definitive evidence for empiric therapeutic anticoagulation without a clear indication Patients with other indications for anticoagulation (e.g. venous thromboembolism or atrial fibrillation) should receive the treatment Risk of hemorrhagic transformation of ischemic stroke Timing of therapeutic anticoagulation initiation, to minimize bleeding risk Limited data on duration of anticoagulation in COVID-19 stroke patients
Poststroke	Telestroke	The extent of virtual care that can be provided to stroke patients Delay to treatment remains a leading cause of post-stroke morbidity and mortality Raising public health awareness to reduce time to hospital presentation and expanding tele-stroke networks to improve time to treatment
	Rehabilitation and Prevention of Secondary Stroke	Less access to physical and occupational therapy services Patients and families tend to discharge to home over inpatient rehabilitation facilities

## Data Availability

Not applicable.
